# The Potential of Different Origin Stem Cells in Modulating Oral Bone Regeneration Processes

**DOI:** 10.3390/cells8010029

**Published:** 2019-01-08

**Authors:** Smaranda Dana Buduru, Diana Gulei, Alina-Andreea Zimta, Adrian Bogdan Tigu, Diana Cenariu, Ioana Berindan-Neagoe

**Affiliations:** 1 Prosthetics and Dental Materials, Faculty of Dental Medicine, “Iuliu Hatieganu” University of Medicine and Pharmacy, Cluj-Napoca, 32 Clinicilor Street, 400006 Cluj-Napoca, Romania; dana.buduru@umfcluj.ro; 2 Stomestet Stomatology Clinic, Calea Manastur 68A Street, 400658 Cluj-Napoca, Romania; office@stomestet.ro; 3 MEDFUTURE—Research Center for Advanced Medicine, “Iuliu-Hatieganu” University of Medicine and Pharmacy, Marinescu 23 Street, 400337 Cluj-Napoca, Romania; diana.gulei@umfcluj.ro (D.G.); zimta.alina.andreea@gmail.com (A.-A.Z.); adrianbogdantigu@gmail.com (A.B.T.); diacenariu@yahoo.com (D.C.); 4 Research Center for Functional Genomics, Biomedicine and Translational Medicine, “Iuliu Hatieganu” University of Medicine and Pharmacy, Marinescu 23 Street, 400337 Cluj-Napoca, Romania; 5 Department of Functional Genomics and Experimental Pathology, The Oncology Institute “Prof. Dr. Ion Chiricuta”, Republicii 34-36 Street, 400015 Cluj-Napoca, Romania

**Keywords:** dentistry, regenerative medicine, stem cells, ESCs, MSCs, iPSCs

## Abstract

Tissue engineering has gained much momentum since the implementation of stem cell isolation and manipulation for regenerative purposes. Despite significant technical improvements, researchers still have to decide which strategy (which type of stem cell) is the most suitable for their specific purpose. Therefore, this short review discusses the advantages and disadvantages of the three main categories of stem cells: embryonic stem cells, mesenchymal stem cells and induced pluripotent stem cells in the context of bone regeneration for dentistry-associated conditions. Importantly, when deciding upon the right strategy, the selection needs to be made in concordance with the morbidity and the life-threatening level of the condition in discussion. Therefore, even when a specific type of stem cell holds several advantages over others, their availability, invasiveness of the collection method and ethical standards become deciding parameters.

## 1. Introduction

Defects in alveolar bone are generally associated with congenital malformations, fractures, trauma events, tumour surgical resection or other surgical procedures and periodontitis [1]. The current standard of care implies the use of dental implants for the recovery of the normal mouth function; even so, not all patients present the desired bone volume for implant procedures. In such cases, the approach consists in autogenous bone grafts for compensation of bone loss [2]. This last procedure is often limited by the surgical stress upon the part of the body where the bone is extracted, amount of extractable tissue that can be collected, possible inflammation and finally resorption of the repurposed bone fragment. The second option, allografts and synthetic materials, are generally poorly osteoinductive or can result in immune rejection [3]. Advances in regeneration medicine have demonstrated the potential of stem cell-based therapies for bone reconstruction in dentistry-associated conditions [4].

**Figure 1:**
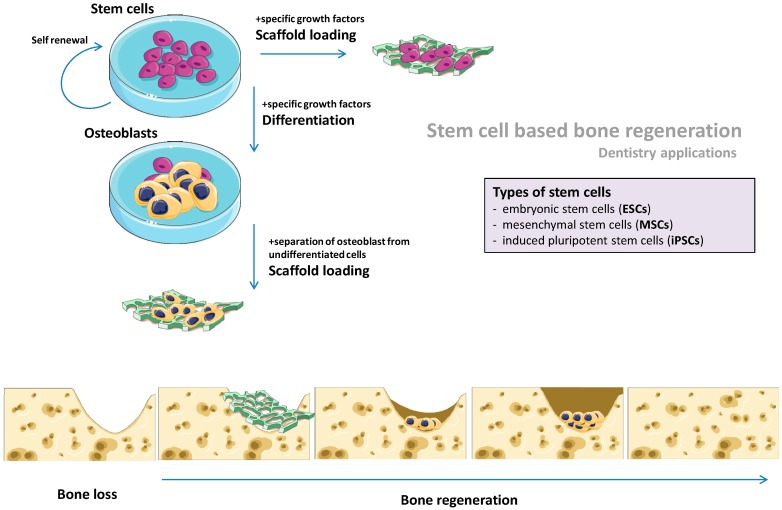
Stem-cell based bone regeneration. Stem cells have the capacity of self-renewal and they can be propagated ex vivo; however, in the presence of specific growth factors with osteogenic potential, the cells differentiate toward osteoblast with the capacity of bone regeneration. For regenerative purposes the cells (undifferentiated or differentiated cells) are embodied in scaffold structures of various biocompatible materials that are further implanted within the affected area.

Regeneration medicine applies to the principle of induced tissue formation for the compensation of pathological loss or malfunction. Most of the time, tissue engineering (referred as regenerative medicine) is based on the potential of stem cells to be artificially directed toward specific cellular phenotypes with the help of opportune environments (e.g., growth factors) [5]. Stem cells of different origins, mainly pluripotent embryonic stem cells (ESCs), undifferentiated multipotent mesenchymal stem cells (MSCs) and induced pluripotent stem cells (iPSCs), are now exploited for the generation of osteoblasts (the prime cells in bone remodelling) (Figure 1).

This review focuses on the different stem substrates used in dentistry regeneration medicine together with their advantages and disadvantages in order to highlight the most opportune strategies for tissue engineering. Additionally, attention is given to different signalling molecules (e.g., cytokines, growth factors) that can positively or negatively affect the osteoblast differentiation. We also present a brief analysis of the scaffold-free approach for bone regeneration, which taps into the intrinsic regenerative capacity of these cells. In the final section, we discuss the possible health risks associated with stem cell therapy and pathological situations that may interfere, highlighting especially the tumorigenic capacity of these cells.

## 2. Types of Stem Cells Used in Regenerative Medicine

**Figure 2:**
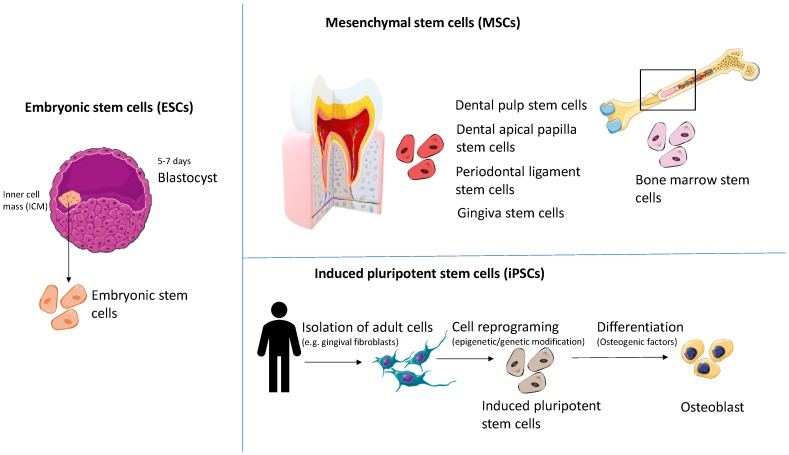
Types of stem cells used for regenerative medicine (focus on bone regeneration). Tissue regeneration is mainly based on three segments of stem cells: embryonic stem cells (ESCs), mesenchymal stem cells (MSCs) and induced pluripotent stem cells (iPSCs). ESCs are isolated from the 5–7 days blastocysts from the inner cell mass and MSCs are harvested from different dental tissues: dental pulp, dental follicle, dental apical papilla, periodontal ligament and gingival or bone marrow. In case of iPSCs, the method implies isolation of adult cells (e.g., gingival fibroblasts) which are subjected to reprogramming mechanisms using epigenetic or genetic modifiers. After the reprogramming strategy, the cells acquire stemness features which allows their further differentiation toward specific cellular entities (osteoblasts) due to the effects of a conditioned media.

Bone regeneration is generally based on the ability of different types of stem cells to reconstitute the adult tissue when subjected to conditioned environments. At present, there are different approaches for tissues engineering that mainly differ in the type of stem cells that is used (Figure 2) or the composition of the differentiation environment. Therefore, we discuss the potential of three main types of stem cells in dentistry bone regeneration strategies, taking into consideration the pros and cons of each stem entity (Table 1).

### 2.1. Embryonic Stem Cells (ESCs)

The use of ESCs for tissue engineering purposes is one of the most efficient method but the controversial debates regarding their provenance are limiting the applications. ESCs are pluripotent stem cells with unlimited self-renewal capacity that can give rise to all types of cells within a human body; their differentiation is programmed (or experimentally programmed) by specific stimulatory factors in order to generate the cell type of interest—e.g., osteoblasts for bone regeneration [6]. ESCs are mainly characterized by the presence of specific markers OCT4, cMYC, KLF44, NANOG, SOX2 [7] which constitute an index of their stemness and regeneration potential.

Despite their significant advantages, there are also other factors to consider for the use of ESCs in regenerative medicine. The main issues are possible tumorigenic characteristics, immunogenicity and also high purity of functional differentiation. Teratomas are tumours originating in germ cells and are often used as a parameter of ESCs pluripotency; formation of such masses is considered as one of the main limitations for the use of ESCs in regenerative medicine. In order to avoid this issue, researchers proposed that the optimal approach in tissue engineering consists of prior differentiation of ESCs toward the desired cell type before transplantation within a living organism. However, also in this case, there is the issue of optimal purity of the cell pool; although techniques like FACS (fluorescence-activated cell sorting) and MACS (magnetically-activated cell sorting) are delivering relatively homogenous cell populations based on specific surface markers expression, the specificity of the markers are still a technical problem. Another direction is represented by the introduction of fluorescence reporter genes within targeted cells for better sorting. In this case, there is a chance of induced tumorigenicity due to chromosomal insertion. Choo et al. applied an opposite approach and used cytotoxic antibodies against undifferentiated cells expressing podocalyxin-like protein-1 (PODXL); however, PODXL is expressed in different adult tissues, indicating the limited specificity of the method [8]. A more direct approach was taken by Mahseni et al., who attempted to target anti-apoptotic genes in cells with pluripotent character in order to limit teratoma formation [9].

Although the use of ex vivo differentiated ESCs was proposed as a safer approach, these terminally-differentiated cells can cause immune responses within the living organism when transplanted for regenerative purposes [10]. In this sense, several strategies have been taken: administration of immunosuppressants, engineering of ESCs to produce immunosuppressive cytokines, short-term blockage of leukocyte-costimulatory factors [11]. ESCs modifications via transfer of a somatic cell nucleus into an egg for somatic cell nuclear transfer (SCNT) [12] and the use of induced pluripotent stem cells (iPSCs). Even when these strategies are successful in minimizing immune reactions upon transplantation of ESCs, the rather invasive nature of the immunosuppressive procedures in patients is not in balance with the actual final output: bone regeneration for dentistry-associated conditions. These factors, together with the limited sources of ESCs are not necessarily sustaining their use in oral bone tissue engineering. ESCs work for bone regeneration it is still in its infancy and no exact conclusion regarding their clinical use can be drawn at this point, especially for dentistry purposes. However, there are several studies that investigated their use for bone regeneration without allocation of specific bone sites (e.g., craniofacial). Early in 2004, Bielby at el. transposed the protocol for murine ESCs and managed to obtain mineralization of osteogenic cells upon manipulation of human ESCs. In vitro propagation of H1 cell line based on murine feeder layers was followed by differentiation via embryoid body (EB) formation; cultivation of the cells isolated from EB in media containing osteogenic additives, especially dexamethasone, concluded in formation of mineralizing cells, positive for osteocalcin and Runx2. Moreover, the ability to replace bone loss was tested in vivo after integration in a poly-D, L-lactide (PDLLA) scaffold; von Kossa staining after 35 days highlighted the presence of mineralized tissue without any tracking of teratoma formation [13]. Despite this apparent success, there was no evidence of actual bone formation; cells that are histological characterized as part of bone tissue—bone-lining cells, osteocytes and bone marrow—were not present in vivo [14]. A more straightforward approach was after applied in order to simulate the actual bone formation. First, a template of cartilage tissue was generated in vitro from mouse ESCs and implanted in immunocompromised mice. Based on this already differentiated scaffolds, in time, there were evidences of lamellar bone formation, positive for osteoblasts, bone-lining cells and osteocytes [15]. However, application of the same protocol for human ESCs was limited and their insufficient chondrogenic potential marked no cartilage presence in vitro or bone in vivo [14]. To overcome these limitations, perfusion culture was applied for the generation of centimetre-size bone grafts from hESC-derived mesenchymal progenitors. The culture conditions involved 3D scaffolds with osteoconductive potential and bioreactors with interstitial flow of media. Once formed, the bone grafts continued to developed within 8 weeks of follow up in immunodeficient models with support vasculature and no signs of teratoma [16]. Another approach involves the formation of bone from human ESCs in the presence of calcium phosphate cement (CPC) scaffolds. Mesenchymal stem cells (MSCs) derived from ESCs and subjected to osteogenic differentiation (increased alkaline phosphatase and osteocalcin) were implanted in vivo under different conditions: supported by CPC alone or CPC in combination with human platelet concentrate (hPC) in different concentrations. Within 12 weeks after in vivo implantation, the groups with hPC showed increased bone and vessel density and osteoblasts presence in comparison with those not stimulated with hPC [17].

### 2.2. Mesenchymal Stem Cells (MSCs)

The oral cavity is a fairly rich source of MSCs with osteogenic potential; stem cells can be found in the dental pulp, dental follicle, dental apical papilla, periodontal ligament [18] and gingiva [19]. Proliferation and bone regeneration capacity is different among these cell types. For instance, in vitro, the apical papilla mesenchymal stem cells (AP-MSCs) are naturally less differentiated than the dental pulp mesenchymal stem cells (DPMSCs) [20].

Stem cells can originate from deciduous or from permanent teeth. In vitro, the DPMSCs from primary teeth have a higher self-renewal capacity than the DPMSC from mature teeth. In addition, they are more capable of osteocyte differentiation and induced mineralization, through elevated levels of endogenous bone morphogenic protein-2 (BMP-2) [21].

Bone marrow MSCs (BM-MSCs) possess the largest osteogenic capacity in vitro, even though DPMSCs have a higher expression of stemness marker OCT-4 [22]. In vitro, BM-MSC and stem cells from synovial tissue have a higher alkaline phosphatase activity and capacity of bone regeneration, than DP-MSCs from adult tooth and from exfoliated decidual teeth [23]. Moreover, the BM-MSC soluble growth factors (GFs) are very efficient in inducing osteogenic differentiation of human dental pulp cells (hDPCs), in an in vitro experimental model [24]. Therefore, BM-MSCs are more suitable for bone regeneration but their harvest implies a relatively invasive procedure with potential risks for the patient [25].

When translating the preclinical results for bone regeneration, researchers have to consider the local environment of the specific site of implantation—oral microenvironment and also take in consideration the condition created upon bone loss—wound site. Therefore, the inclusion of preclinical models that closely mimic the in vivo context of the actual patient by mirroring the local microbiota, modulatory factors (like cytokine releasement, growth factors, heterogeneous cell interaction and biological fluid income—e.g., saliva) is mandatory. The oral microenvironment affects the behaviour of stem cells and their bone-regenerative potential. For instance, an in vitro experiment proved that BM-MSCs and DPMSCs have a decreased osteogenic capacity when the local pH is low. This usually happens in case of inflammation caused by an underling pathology [26]. Patients with periodontics have a decreased number of stem cells in the oral cavity. In addition, these cells have a lower capacity of regeneration due to the inflammatory conditions which characterize the local environment. Therefore, a group of researchers proposed the enhancement of the regeneration capacity for periodontal ligament stem cells (PDLSCs) by inserting into the cells the recombinant human IGFBP5 protein (rhIGFBP5). This led to a higher osteogenic differentiation capacity in vitro and in an animal model of inbred male minipigs [27].

The harmful effects of the pro-inflammatory microenvironment affect in particular stem cells in the oral cavity, whereas BM-MSCs are more resistant in in vitro conditions [28]. When cultivated in vitro in the presence of tumour necrosis factor alpha (TNFα)-enriched media, PDLSCs exhibit decreased levels of Runx, a marker of bone formation, in comparison with BM-MSC. Also the mineralization capacity of BM-MSCs is larger than that of PDLSCs, based on lower alkaline phosphatase (ALP) activity [29].

The presence of Gram-negative bacteria and their lipopolysaccharides (LPS) in mouth of adult male Sprague–Dawley rats and wound sites also decreases the osteogenic potential of PDLSCs through binding of Toll-like receptor 4 (TLR4) and activation of the NFκB pathway; whereas this does not happen in the case of BM-MSCs [30].

In order to enhance bone regeneration capacity of oral stem cells, it has been proposed to co-culture oral stem cells with BM-MSCs. The in vitro co-culture of two types of MSCs, BM-MSCs (from jaw bone) and PDLSCs, is more effective in emulating the 3D bone tissue organization (e.g., ratio and network architecture of Sharpey-like fibres and collagen fibres) compared to monocultures [31]. BM-MSCs derived from maxilla enhance the osteogenesis capacity of PDLSC, in vitro [32]. The BM-MSCs have an elevated in vitro capacity of osteogenesis when treated with zinc ions as is shown by elevated levels of type I collagen, alkaline phosphatase (ALP), osteocalcin (OCN) and Runx2 expression [33].

An adequate scaffold design can modulate local inflammation and surpass the limitations of MSCs from oral cavity. The periodontal ligament cells (PDLCs) and the BM-MSCs have been inserted into a calcium alginate hydrogel. Both types of stem cells were effective in inducing osteogenic differentiation and the scaffold led to a lower degree of inflammation at the local tissue, in vivo, in New Zealand rabbits [34]. The octacalcium phosphate ceramic (OCP) granules offer the same degree of osteogenic differentiating capacity in the case of gingiva-derived mesenchymal stromal cells (GMSCs) and BM-MSCs, in nude mice [35]. GMSCs are also competent in osteogenic differentiation although their capacity was assessed only in a few number of studies. The Enamel matrix derivative (EMD) helps differentiation of GDMSCs, in vitro [36].

In an in vitro experimental scenario, PDLSCs co-cultured with induced pluripotent stem cells (iPSCs) derived from adipocytes enhance expression of osteogenic markers in iPSCs [37]. The alveolar bone marrow-derived MSCs (aBMSCs) may represent a source of less invasive BM-MSCs, which keeps the osteogenic potential of the BM-MSCs in vivo, in a mouse model [38].

For regeneration of bone tissue, stem cells kept at the stem level and implanted in vivo, in male Fischer rats, as such, are less effective in bone regeneration than stem cells directed toward osteogenic differentiation before implantation. The chondrocyte differentiation is also an option for the replacement of the bone alveolar sack but it is less effective than the osteogenic differentiation [39].

Investigation of bone-regenerative potential of stem cells is still in its infancy. The methods used at present are informative but lack standardization of procedures, as a recent meta-analysis stated [40] and progression is still slow. Many studies focus on exploring regenerative techniques without too much attention to ethical aspects or considerations whatever the procedure will ever be translated in clinical practice in human patients. MSCs can have different molecular mechanism to induce osteogenesis. In a recent in vitro study, inhibition of the Wnt signalling stimulated bone differentiation of PDLSCs under inflammatory conditions, whereas in the case of BM-MSCs, osteogenesis was impaired [28]. DPSCs can induce immune tolerance by increasing numbers of local regulatory T cells (Treg) in vitro and in mouse alloskin graft model [41].

### 2.3. Induced Pluripotent Stem Cells (iPSCs)

Induced pluripotent stem cells (iPSCs) offer a great potential for tissue regeneration. This is reasoned by the fact that, in an adult organism, the availability and number of differentiated cells is greater than that of the stem cells. In addition, these stem cells are easily replaceable in the case of regeneration failure. This therapeutic approach avoids a number of ethical issues that usually surround the use of stem cells in regenerative medicine [42]. For instance, in in vitro conditions, iPSCs show a similar capability of bone regeneration as human umbilical MSCs when encapsulated to hydrogel fibres [43]. The growth/differentiation factor-5 (GDF-5) coupled with hydrogel enhances osteogenesis in the case of iPSCs from dental tissue better than BM-MSC, in an murine model [44].

The process of epigenetic reversal of differentiation with the scope of regaining pluripotent characteristics in adult cells is called reprogramming [45]. Once the cells are reprogrammed to become stem cells, they can be again differentiated into another cell type, such as osteocytes. This is done through the activation of the Wnt/β-catenin signalling pathway [46] or Notch signalling pathways, which function as an *on and off* switch for osteogenic differentiation in the case of iPSCs from gingival fibroblasts, in vitro [47]. The process of cell reprogramming can take place in vivo, at the wound site or ex vivo, in the culture dish.

iPSCs are integrated into a scaffold which has additional conditioning factors to direct the gain of certain differentiated features. In an in vitro study, fibroblasts from gingival tissue were reprogrammed to become stem cells with the help of OCT3/4, SOX2, KLF4 and c-MYC transduction. Afterwards, the stem cells were inserted into a titanium disc and conditioned with osteogenic media. The formation of osteogenic cells was observed after 28 days [48].

iPSCs are usually conditioned to become osteogenic with the help of growth factors (GF), such as bone morphogenetic protein- 2 (BMP-2), BMP-7, transforming growth factor-β (TGF-β) and fibroblast growth factor (FGF) [49]. A complex of hydrogel-bone morphogenetic protein-6 (BMP-6) and iPSCs from rat fibroblasts was tested on rats with maxillo-molar defects; after 6 weeks an enhanced regeneration capacity was found [50]. The lentiviral transduction of BMP-2 is more effective in promoting osteogenic differentiation and matrix mineralization, in vitro [51]. An alternative to GFs are the inorganic compounds, such as the inorganic phosphate polymer which activates the Wnt/β-catenin signalling pathway [52].

The vascularization of a newly formed bone tissue is of equal importance as the actual formation. A study has shown that a calcium phosphate cement (CPC) scaffold containing three type of cells: pericytes, vascular endothelial cells and iPSCs is more effective in promoting a successful bone regeneration in vivo, in male athymic nude rats, than scaffolds containing only one or two of the three cell types [53].

The capacity of mature cells to become stem cells varies and depends mainly on the self-renewal capacity of future iPSCs. For instance, gingival fibroblasts can resist up to 50 passages in culture as opposed to dermal fibroblasts which can resist only a few passages. This makes gingival fibroblasts a better alternative when it comes to the selection of mature cells for reprogramming in a mouse in vivo model [54].

iPSCs derived from neural crest cells (NCCs) have a superior regenerative capacity in the case of craniofacial wounds, because during development, NCCs help the formation of major types of cells. Through activation of Wnt signalling and silencing of TGF-beta signalling, NCCs can be differentiated to MSCs. NCC-MSCs have a distinct genetic signature. The ACKR3, L1CAM, PTPRB, MEIS2 and ANKRD1 genes were significantly overexpressed in NCC-MSCs in comparison to BM-MSCs, PDSCs, DPSCs and NCCs, in vitro [55]. The use of iPSCs for the repair of oral bone defects, mainly alveolar bone has long been proposed. For instance, a comparison between the use of simple silk or email scaffolds with the use of combined scaffold with iPSCs revealed that, while the simple scaffolds may induce some-osteogenic related genes, while repressing the others, in the case of iPSCs, all the relevant osteogenic genes were up-regulated: OC, Osx and Runx2 [56]. A complex strategy containing BMP-6-hydrogen combined with iPSCs minimized the inflammation and stimulated the tissue mineralization of bone maxillary-molar defects in rats [50]. In a periodontitis mouse model, the bone loss due to chronic and acute inflammation was decreased in the case of mouse iPSCs inoculation [57]. The origin of iPSCs may be involved in their regenerative capacity, considering that iPSCs from periodontal ligament are more effective in regenerating the periodontal tissue as opposed to iPSCs from neuronal crest or iPSCs from skin fibroblasts [58]. iPSCs were loaded on an email-derived extracellular matrix combined with atelocollagen sponge, both known to have oral bone regenerative capacity. The complex exhibited higher expression of the bone markers sialoprotein and osteopontin, after 14 days post in vitro culture [59]. iPSCs have similar potency to BM-MSCs in the case of cranial new bone formation and angiogenesis, when co-cultured with human endothelial cells on a calcium phosphate cement scaffold [60].

Media content is a crucial factor in manipulating the osteogenic potential of cells; fibrinogen induces osteogenic differentiation of a human embryonic stem cell line. After in vitro fibrinogen treatment, cells exhibit increased levels of osteopontin, osteocalcin, Runx2 and osterix, whereas expression of stem markers is decreased [61]. iPSCs from mouse gingival fibroblasts were cultured in a 3D model and programmed toward osteogenic differentiation through the application of a hydrogel, calcium, phosphorous and hydroxyapatite. The cells were then implanted in vivo and induced bone formation. However, at the same time this scaffold-free construct also induced the formation of teratoma tissue around the wound area [62]. On the other hand, iPSC from human were inserted into a self-assembly peptide hydrogel and then implanted at the cranial wound site in rat calvarial bone defect. The scaffold complexed with iPSCs was the most effective in repairing the bone wound in comparison to the naked scaffold or saline [63].

The use of adult cells for reprogramming purposes has the potential to overcome much of the ethical, technical and political obstacles; more research is needed to obtain reliable, reproducible and efficient reprogramming strategy. Furthermore, microRNAs (miRNAs), small non-coding sequences intensively explored in cancer therapy and diagnosis [64], are able to modulate the pluripotency of genes. Therefore, by only inducing the expression of a single miRNA cluster (miR-302/367 cluster), Anokye-Danso et al. managed to reprogram somatic cells at a rate of 100-fold more efficiency than in the case of classical strategies—OSKM transcription factors [65]. Additionally, genome editing techniques, like CRISPR/Cas9 [66] hold great potential with respect to iPSCs for personalized therapeutic regenerative medicine [67]. Recently, Brunger et al. designed inflammation-resistant murine iPSCs by altering the IL-1 signalling with the help of CRISPR/Cas9 targeted toward interleukin 1 receptor 1 (Il1r1). Cells differentiated into cartilage tissue were exposed to three day treatment with 1 ng/mL IL-1α and presented resistance to pro-inflammatory environments [68]. As previously stated, oral cavity inflammatory conditions decrease the regenerative potential of stem cells, where this strategy could work in generating iPSCs resistant to such stressful conditions. Although applied for cartilage formation, this study can be translated for bone regenerative strategies. Adenoviral mediated gene therapy for reinforcement of BMP-2 and Pluronic F127 (PF127) genes in autologous bone marrow mesenchymal stem cells successfully conducted to the regeneration of periodontal attachment apparatus that includes also the alveolar bone [69]; this application can hold great potential in CRISPR/Cas9 based approaches for shift of iPSCs toward increased osteogenic potential.

## 3. Stem Cells-Based Scaffold-Free Bone Regeneration

An alternative method has been developed to culture stem cells. The cell sheet construction, which is meant to keep cells stacked together and in close contact with each other, allows them to form thigh junctions, facilitating the exchange of information and keeping an integrated basal lamina. This approach has been proven to have superior characteristics compared to cells on a scaffold [81]. The DPSC cell sheet remains viable until 72 h, then reach a plateau. The cell sheet exhibits the same level of mesenchymal markers as cells on a scaffold, with the exception of OCT3/4, which was higher expressed in the case of cells in a sheet and STRO1 which was less expressed in cells in a sheet. The cell sheet also exhibits a larger mineralization potential [82].

The cell sheet is also an effective delivery method for the enhancement of regenerative potential with help of miRNA therapy. BM-MSCs cell sheet was wrapped around a titanium implant and cells were transfected with anti-miR-138. In an in vivo model of immunocompromised mice, the cells showed a stable downregulation of miR-138 locally (at the site of the implant) and enhanced the bone regenerative process. At the molecular level, ALP, OCN, RUNX2 and OSX were expressed at higher levels than non-transfected cells [83]. In an in vivo model of female domestic pigs, BM-MSCs were harvested and cultured as a 3-layer cell sheet. The cells showed enhanced mineralization levels and regenerative capacity. As control, bone without treatment was taken. The side with the cell sheet was thicker and the bone volume was larger [84].

The cell sheet approach was also used with miR-122. BM-MSCs were cultured to form a cell sheet and then transfected with miR-122-containing vectors. Transfected cells showed enhanced capability of osteogenesis. Expression of early osteogenic biomarkers, ALP and COL-1 and of late bone formation biomarkers, OCN and BMP-2, was also increased in case of transfected cells. Moreover, p-ERK was detected at protein level only in the case of transfected cells. The osteogenic potential was kept on the long term. After 8 weeks post-transfection and in vivo implementation, in rats, the bone gaps were smaller and the tissue had a better developed angiogenic network [85]. The combination of BM-MSCs and endothelial progenitor cells (EPCs) induced more rapid bone formation in case of alveolar bone in vivo on ovariectomized rat [86].

Another option for the construction of a scaffold is a decellularized scaffold. In a recent experiment, the scaffold was populated with periodontal ligament MSCs, after which the scaffold was decellularized. The scaffold was again populated with PDL-MSCs or with MSCs from umbilical tissue. Both types of MSCs were capable to induce bone regeneration. BM-MSCs were used to wrap a titanium structure with a PDL-MSC cell sheet. This complex was more capable of cementum formation, which is an advantage in the case of bone formation, due to its protective effects against infection and increased bone regeneration capacity, in vivo, in the case of canine mandibular bone [87].

The decellularized structure had a higher regenerative potential. The osteogenic and angiogenic markers, BMP2 and osteocalcin and VEGF, respectively, exhibited increased expression levels throughout the period of evaluation. STRO-1, a marker of a mesenchymal phenotype, showed decreased expression from day 7 to day 14 and then its expression was undetectable in the case of the decellularized constructs in comparison to the classical scaffold option, in rat periodontal defect [88].

A better option to cells sheets may be cell constructs in 3D culture of MSCs which can be differentiated or not prior to implantation. The result of this procedure is called a 3D construct. A study compared the osteogenic potential of the cell sheet and the bone construct from DPSCs and showed that the cell construct was more effective in inducing oral bone formation. In vitro, the differentiated construct showed the highest expression of bone biomarkers at the mRNA and protein level—osteopontin (OPN), bone sialoprotein (BSP), osteocalcin (OCN) and type I collagen (Col 1)—when compared to the sheet or to the undifferentiated cells [89].

Dental pulp cells were grown in a similar construct and after 3 months of in vivo implantation cells showed increased thickness of bone-like tissue [90]. PDL-SCs tends to form mainly periodontal tissue in vivo, whereas the BM-MSCs form predominately bone structures. A study attempted to form a complex composite of fibrin scaffold containing growth factors, PDL-MSCs and BM-MSCs. The complex was effective in inducing both types of structures equally important for a complete regeneration at a dental wound site [91].

Both DPSC and BMSCs cell sheets, when treated with helioxanthin have a more potent oral bone regeneration capacity, compared with untreated cells. However, at the molecular level the stem cell types showed a different expression pattern. On day 14 post treatment, when comparing BMSCs and DPSCs, Alp was overexpressed in BMSCs and osteocalcin was up-regulated in DPSCs [92]. The co-culture of two types of stem cells constitutes a viable option for the building-up of complex oral tissue structures, such as the periodontal structure (contains also bone tissue). A study concluded that the cell sheet from jaw BMSCs and PDLSCs is more efficient in regenerating bone architecture, motivated by the overexpression of bone related genes ALP, RUNX2, BSP, COL-1, OCN, integrinβ1, fibronectin and periostin in the case of co-culture versus single-culture [31]. The BMSC cell sheet, cultured in normal conditions, with dexamethazone and ascorbic acid phosphate, was implanted at the mandibular wound site in a rat model of oral bone injuries. The cell sheet was effective in inducing bone regeneration at a fast pace [93].

However, controversies remain regarding the single use of cell sheet. The platelet-rich fibrin (PRF) implanted together with the adipose-derived stem cells (ASCs) cell sheet, showed a higher proliferation and oral bone differentiation capacity, in comparison with only ASCs cell sheet, only PRF or no treatment [94].

The search for the intrinsic capability of stem cells to induce bone formation has to be encouraged, because it will strongly reduce the toxicities associated with scaffolds. The present data shows that these types of approach have an even better therapeutic effect.

## 4. The Osteogenic Potential of Stem Cells and Its Correlation with Various Pathologies—Focus on Mesenchymal Stem Cells

The major concern when it comes to using regenerative therapy in the everyday clinical practice consists in danger of developing cancer. This is a logical conclusion and a widely recognized issue when considering the many similarities between stem cells and malignant cells [95]. The studies on this aspect are somehow divided, where a number of research have revealed that MSCs have inhibitory effects upon malignant formation [96,97,98], being counteracted by similar studies where the tumour promoting role of MSCs are highlighted [99,100]. MSCs are chemoattracted to tumour sites [101]; also are found in a greater number in the case of head and neck tumours, in comparison to healthy tissue. The cells are capable of differentiating in tumour-associated fibroblasts which leads to the formation of new blood vessels or acquired immunosuppressant features [102].

Oral cancer cells are similar to iPSCs, by going through a reprogramming process and expressing at the end the stem markers Oct3/4, c-Myc, Sox2 and Klf4 [103]. Moreover, iPSCs are often considered to be a dangerous choice of stem cells which can go through malignant transformation due to their genomic instability [104].

The data is still conflicting. A recent study has demonstrated that MSCs derived from normal gingival tissue secrete soluble factors which inhibit the development of oral cancer. At the molecular level, overexpression of the pro-apoptotic genes p-JNK, cleaved PARP, cleaved caspase-3 and Bax was observed and down-regulation of anti-apoptotic genes: p-ERK1/2, Bcl-2, CDK4, cyclin D1, PCNA and survivin [105].

An important aspect in predicting the tumorigenicity of stem cells consists in the immune status of the preclinical models. In this sense, immune deficient animals may not be suitable for evaluation of tumour formation upon MSCs (or stem cells in general) administration; it was shown that the shift toward immune competent models allogeneically transplanted with MSCs prevented the formation of cancer due to immune rejection of the allogenic cells [95]. MSCs were applied in first human study on the alveolar bone and it led to enhanced bone regeneration and no local or systemic side effects [106]. Moreover, generally, no malignancy has been yet diagnosed in clinical trials that involved the administration of MSCs [95].

The osteogenic capacity of stem cells is affected by systemic conditions in the recipient organism. Still, DPSCs or GMSCs from inflamed tissues had a higher proliferation rate and expressed the cytoskeleton markers, profilin-1, cofilin-1 and vinculin and heat shock proteins (HSP90 and HSPA9) at higher levels than MSCs from healthy teeth [107].

**Figure 3:**
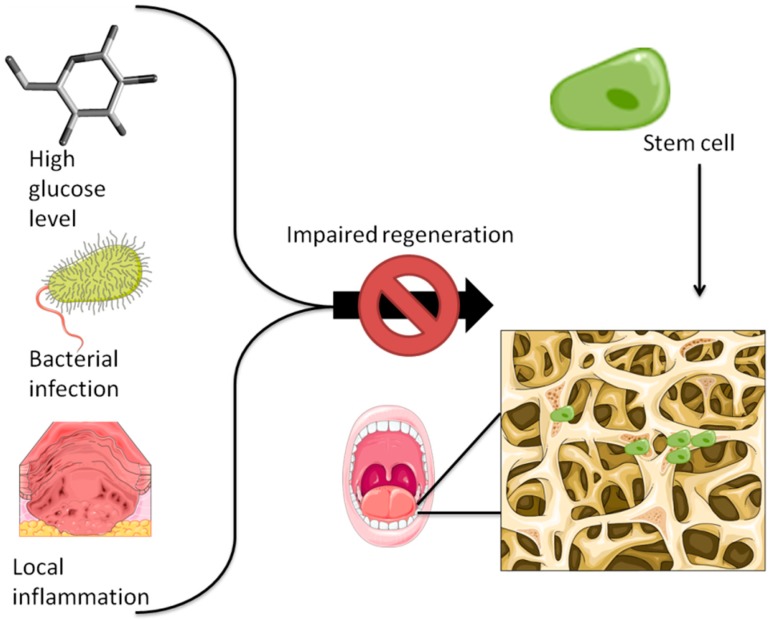
The capability of stem cells to induce bone regeneration is affected by the local microenvironment. Damaging factors include a high concentration of glucose (specific for diabetes), the presence of harmful bacteria and the local inflammation present at the wound site.

AP, osteocalcin, Runx2, VEGF and angiopoietin were upregulated in the case of BM-MSCs exposed to an environment rich in pro-inflammatory cytokines [108]. Further details about the effects of pathological states in modulating the capacity of bone formation of stem cells are found in Table 2 and are illustrated in Figure 3.

## 5. Conclusions

Tissue engineering holds great potential in regenerative medicine with high impact in critical fields including organ transplantation for life threatening disease; this great segment of applications includes also bone reconstruction for dentistry-associated conditions where the bone volume affects the normal function of the oral cavity. At the moment, there are different strategies for bone regeneration that take in consideration a large plethora of stem cells with different differentiation potentials. In the process of decision making, researchers and clinicians should carefully balance the effectiveness of different stem cells with their availability and invasiveness of the collection method with the impact of the condition/pathology for which are used. Therefore, under an objective perspective, for dentistry regeneration purposes, iPSCs seem to be the most appropriate type of cells. This affirmation is mainly relying on the minimal invasiveness of the harvesting method and high availability. Even so, there are concerns regarding the tumorigenic and immunogenic potential of iPSCs due to multiple genetic or/and epigenetic manipulation and also high associated costs. For future perspective, the long term storage of stem cells from deciduous teeth after the natural replacing processes could represent an optimal option.

## Figures and Tables

**Table 1:** Characteristics of different types of stem cells used for regenerative medicine.

Type of Stem Cells	Source	Harvesting Discomfort	Standardization—Study Level	Osteogenic Potential	Self-Renewal Capacity	Costs	Ethical Conflict	Availability	Translational Level	Ref.
ESC	Blastocyst	N/A	+	+++	+++	+++	+++	+	+	[70,71,72]
BM-MSC	Bone marrow from iliac crest, jaw, maxilla	+++	+++	++	+	++	++	+	++	[73]
PDLSC	Periodontal ligaments (wisdom teeth)	++	+	+	+++	+	+	+	+	[74,75,76]
DPMSC	Dental pulp from primary or permanent teeth	+	++	+	+	+	++	+	+++	[76,77]
GMSC	Connective tissue from gingiva	+	+	+	++	+	+++	+	++	[77,78]
iPSC	Adult cells, especially gingival fibroblasts	+	+	++	+	+++	++	+++	+	[70,71,72,79,80]

+ minimal; ++ medium; +++ high; N/A not available.

**Table 2:** The effect of pathological states on the capacity of bone regeneration of stem cells.

Condition	Type of Stem Cell	Effect	Ref
High glucose	PDL-MSC	Suppressed proliferation and differentiation into osteoblasts	[109]
High glucose	DPSCs	Impaired proliferation and differentiation	[110]
Inflammation	BM-MSCs	Enhanced capacity	[28]
Inflammatory conditions	PDLSCs	Impaired osteogenic differentiation	
Inflammatory conditions	BM-MSCs	Normal osteogenic capacity	
Inflammation	BM-MSCs coupled with titanium implants	Stimulated bone formation but disorganized tissue	[111]
Inflammation	DPSCs	Anti-inflammatory effect	[41]
Inflammation—NFkB expression	DPSCs	Down-regulated NfKB signalling lead to increased osteogenic potential	[112]
Infection with Porphyromonas gingivalis	PDL-MSC	Osteoblastic differentiation and promotion of pro-inflammatory cytokine production	[113]
Exposure to lipopolysaccharides	PDL-MSC	Does not affect stem cell markers	[114]
Bone loss in lupus erythematosus	BMMSCs	Systemic administration reduced Il-17 level and recovered bone loss	[115]
	DPSCs	Recovered bone loss	
Autoimmunity	DPSCs	Increased Tregs and decreased TH17; are capable of osteogenic differentiation	[116,117]
	BM-MSCs	Osteogenesis	
